# Isolation of Efficient Xylooligosaccharides-Fermenting Probiotic Lactic Acid Bacteria from Ethnic Pickled Bamboo Shoot Products

**DOI:** 10.3390/biology11050638

**Published:** 2022-04-21

**Authors:** Apinun Kanpiengjai, Pongsakorn Nuntikaew, Jirat Wongsanittayarak, Nalapat Leangnim, Chartchai Khanongnuch

**Affiliations:** 1 Division of Biochemistry and Biochemical Innovation, Department of Chemistry, Faculty of Science, Chiang Mai University, Chiang Mai 50200, Thailand; pongsakornbigc@gmail.com (P.N.); panatnant_n@cmu.ac.th (J.W.); 2 Research Center of Microbial Diversity and Sustainable Utilization, Faculty of Science, Chiang Mai University, Chiang Mai 50200, Thailand; 3 Research Center for Multidisciplinary Approaches to Miang, Chiang Mai University, Chiang Mai 50200, Thailand; chartchai.k@cmu.ac.th; 4 Graduate Program in Biotechnology, The Graduate School, Chiang Mai University, Chiang Mai 50200, Thailand; nalapat_l@cmu.ac.th; 5 Division of Biotechnology, School of Agro-Industry, Faculty of Agro-Industry, Chiang Mai University, Chiang Mai 50100, Thailand

**Keywords:** pickled bamboo shoot, probiotic, lactic acid bacteria, xylooligosaccharides, xylanolytic

## Abstract

**Simple Summary:**

Naw Mai Dong is an ethnic fermented bamboo shoot product that is popular in the upper northern region of Thailand. Xylan is a component of hemicellulose that can be found in bamboo shoots. It is a raw material utilized in xylooligosaccharide production. To produce Naw Mai Dong, bamboo shoots are sliced and pickled in bottles or jars that contain water rinsed from rice crops with or without salt. This would differ from the simple brine comprised of water and salt that is associated with most other pickled products. Naw Mai Dong is sour due to its acidity. Importantly, fermented fruits and vegetables are a potential source of lactic acid bacteria, which have been well-documented to possess a number of probiotic characteristics. This would imply that some of the lactic acid bacteria present during bamboo shoot fermentation could serve as efficient xylooligosaccharides-fermenting probiotic lactic acid bacteria.

**Abstract:**

Xylooligosaccharides (XOSs) are produced from xylan, which is a component of the hemicellulose that can be found in bamboo shoots. Naw Mai Dong, an ethnic pickled bamboo shoot product of northern Thailand, is generally characterized as acidic and has a sour taste. It can be considered a potential source of probiotic lactic acid bacteria (LAB). This study aimed to isolate efficient XOSs-fermenting probiotic LAB from ethnic pickled bamboo shoot products. A total of 51 XOSs-fermenting LAB were recovered from 24 samples of Naw Mai Dong, while 17 strains exhibited luxuriant growth in xylose and XOSs. Among these, seven strains belonging to *Levicaseibacillus brevis* and *Pediococcus acidilactici* exhibited similar growth in glucose, xylose, and XOSs, while the rest showed a weaker degree of growth in xylose and XOSs than glucose. Sixteen strains exhibited resistance under gastrointestinal tract conditions and displayed antimicrobial activity against foodborne pathogens. Notably, *Lv. brevis* FS2.1 possessed the greatest probiotic properties, with the highest %hydrophobicity index and %auto-aggregation. Effective degradation and utilization of XOSs by probiotic strains are dependent upon xylanase and β-xylosidase production, as well as xylose metabolism. It can be concluded that pickled bamboo shoot products can be a beneficial source of XOSs-fermenting probiotic LAB.

## 1. Introduction

Xylooligosaccharides (XOSs) are oligosaccharides composed of 2–10 xylose residues that are linked through β-(1,4) bonds. They can be produced via the enzymatic hydrolysis of xylan [1]. After being recognized as important dietary fibers, one of the most significant features of XOSs as a food ingredient is their potential to be used as a prebiotic in enhancing the growth of intestinal beneficial flora, particularly *Bifidobacterium* spp. [2]. In addition, various species of *Lactobacillus* sp. have been extensively investigated for their XOSs-fermenting ability, such as *Lb. acidophilus*, *Levilactobacillus brevis* (new name of *Lb. brevis*), *Lb. casei*, *Lb. crispatus*, *Lb. delbrueckii*, *Limosilactobacillus fermentum* (new name of *Lb. fermentum*), *Lb. gasseri*, *Lb. johnsonii*, *Lactococcus lactis*, *Lb. mucosae*, *Lb. pentosus*, *Lactiplantibaillus plantarum* (new name of *Lb. plantarum*), *Lacticaseibacillus rhamnosus* (new name of *Lb. rhamnosus*), and *Lb. sakei*. However, there have only been a few reports of probiotic strains with effective XOSs-fermentation capabilities [3]. The microbial metabolism of homologous XOSs depends upon xylanase, β-xylosidase [4], and the ability to metabolize xylose [5], while the dependencies of those same enzymes that are produced by other probiotic LAB have yet to be established. The ability to metabolize XOSs in vitro may be a rare trait among LAB. This determination may limit the application of LAB and XOSs as functional food ingredients.

**Figure 1:**
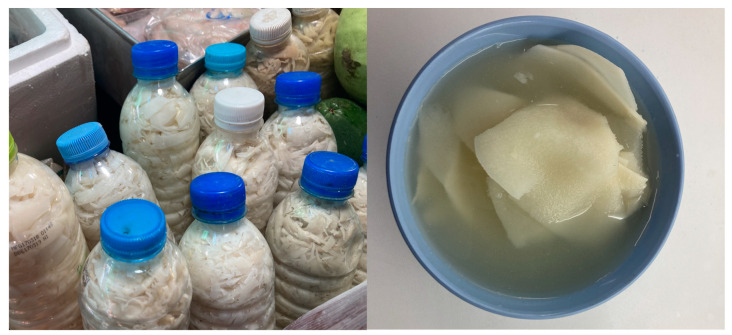
Appearance of Naw Mai Dong commercially available in a local market.

Naw Mai Dong is an ethnic fermented bamboo shoot product that is popular in the upper northern region of Thailand (Figure 1). The annual mass production period takes place in the early rainy season (May to July), when bamboo shoots are generally abundant. Bamboo shoots are collected from the edges of rice fields, around people’s homes, in the woods, and up on hills. They are sliced thin and pickled in bottles or jars that contain water rinsed from rice crops with or without salt, making it different from the simple brine comprised of water and salt associated with other pickled products. Fermentation is carried out at ambient temperatures for at least three days. The resulting product is sour due to its acidity, with pH values ranging from 4 to 5.5. The chemical composition of bamboo shoots primarily consists of cellulose and hemicellulose, respectively [6]. Since xylan is a major component of hemicellulose, this would imply that the microorganisms that are associated with bamboo shoot fermentation may be able to efficiently degrade and utilize xylan and its oligomers (XOSs) through their xylanolytic enzyme-producing ability. With regard to the acidity of the final pickled bamboo shoot product, it is entirely possible that acid-tolerant microorganisms could be employed as probiotics. In addition, it is expected that pickled bamboo shoot products may selectively provide conditions for the selective enrichment of XOSs-fermenting probiotic LAB. The aims of this research study were to isolate efficient XOSs-fermenting LAB and evaluate probiotic properties of the isolated LAB in vitro for further food applications. The enzymes associated with XOSs degradation were also identified.

## 2. Materials and Methods

### 2.1. Chemicals

Analytical-grade xylose was purchased from Loba Chemie (Mumbai, India). Food-grade XOSs for culture medium preparation were obtained from Triple Nine Solution Co. Ltd. (Bangkok, Thailand). Food-grade XOSs were confirmed to be free of xylose contamination before being used. Standard XOSs, including xylobiose (X2), xylotriose (X3), xylotetraose (X4), and xylopentaose (X5), were purchased from Megazyme (Wicklow, Ireland). The nutrient medium, trypticase soy broth, and Columbia blood agar were purchased from HiMedia (Nashik, India). The pepsin, bile salt, and pancreatin used in the preparation of simulated gastrointestinal tract (GIT) conditions were obtained from Sigma-Aldrich (St. Louis, MO, USA).

### 2.2. Microorganisms and Culture Conditions

Bacterial pathogens, including *Bacillus cereus* TISTR 747, *Escherichia coli* TISTR 527, and *Salmonella enterica* ser. Thyphimurium TISTR 1472, were cultured in a nutrient broth (NB), while *Listeria monocytogenes* DMST 17303 was cultured in trypticase soy broth (TSB). The first four bacteria were grown aerobically on nutrient agar (NA) at 37 °C. On the other hand, *L. monocytogenes* was grown anaerobically on trypticase soy agar (TSA) in an anaerobic jar at 37 °C. Isolated XOSs-fermenting LAB were cultured in modified deMan Rogosa and Sharpe broth (mMRSB) (10 g/L peptone, 10 g/L beef extract, 5 g/L yeast extract, 2 g/L di-ammonium hydrogen citrate, 5 g/L sodium acetate, 1 mL/L tween80, 0.2 g/L MgSO_4_∙7H_2_O, 0.2 g/L MnSO_4_∙H_2_O, and 10 g/L carbon source) containing 10 g/L XOSs (mMRSB-XOSs) as the sole carbon source. When necessary, each bacterium was static-cultured in the mMRSB-XOSs at 37 °C for 24 h and then plated on mMRS agar (mMRSA) containing 10 g/L XOSs as the sole carbon source (mMRSA-XOSs) using the streak plate technique. 

### 2.3. Sampling Locations and Sample Collection

The sampling sites for pickled bamboo shoot products were located in six provinces of northern Thailand, including Chiang Mai, Nan, Phrae, Chiang Rai, Phayao, and Lampang. All samples were collected during the period from May to July 2020.

### 2.4. Enrichment and Isolation of Xylose-Fermenting LAB

Approximately 5 g of bamboo shoot products were homogenized in 10 mL of sterile 0.85% NaCl as a diluent using a Masticator^®^ homogenizer blender (Barcelona, Spain) for 5 min. The homogenate was then transferred to a laboratory bottle containing 40 mL mMRSB-XOSs. An enrichment step was carried out at 37 °C for 24–48 h. The culture that exhibited optical density at 600 nm (OD_600_) of more than 1.00 was selected for the isolation of LAB. The turbid culture was serially diluted from 10^0^ to 10^5^ using a diluent. Accordingly, 0.1 mL of each dilution was then plated on mMRSA-XOSs supplemented with 125 ppm of bromocresol purple and then incubated at 37 °C for 48 h. The distinct colonies that presented clear yellow zones around the colonies, on a purple background, were isolated and streaked on the same medium for the purposes of purification. They were assumed to be XOSs-fermenting LAB. The yellow clear zone produced on mMRSA-XOSs was associated with the hydrolysis of XOS in releasing fermentable sugars for the production of short-chain fatty acids, i.e., lactic acid and acetic acid [7].

### 2.5. Confirmation and Screening for XOSs-Fermenting LAB

XOSs-fermenting LAB were grown in a screw-cap tube containing 10 mL of mMRSB-XOSs at 37 °C. After 24 h of cultivation, the cells were harvested by centrifugation, washed twice with phosphate buffer saline (PBS) solution, and resuspended in the same solution to a final OD_600_ of 0.5. The resulting cell suspension was used as an inoculum. A total of 10% (*v*/*v*) of inoculum was transferred to a screw-cap tube containing 10 mL of two different media comprised of mMRSB-X (mMRSB containing 10 g/L xylose as the sole carbon source) and mMRSB-XOSs. The mMRSB containing 10 g/L glucose was used as a control. After being incubated at 37 °C for 24 h, the culture was measured for OD_600_ prior to cell removal by centrifugation. The supernatant was collected for pH measurement and the determination of residual carbohydrates using the phenol–sulfuric acid method [8].

### 2.6. Bacterial Identification

The genomic DNA of LAB was extracted using a Wizard^®^ Genomic DNA purification kit (Promega Corp., Madison, WI, USA) according to the manufacturer’s instructions. The 16S rRNA gene was amplified with the use of a set of primers, namely 27F (5′-AGAGTTTGATCCTGGCTCAG-3′) and 1492R (5′-GGTACCTTGTTACGACTT-3′), while the genomic DNA was used as a template according to the protocol previously described [7]. The amplicon of the 16S rRNA gene was purified and sequenced using a sequencing service provider (1st BASE Pte Ltd., Singapore). For bacterial identification, pairwise alignment of the 16S rRNA gene sequence was performed using the standard nucleotide blast tool offered by the National Center for Biotechnology Information (NCBI) available at https://blast.ncbi.nlm.nih.gov/Blast.cgi (accessed on 14 December 2021). The neighbor-joining method employing bootstrap resampling from 1000 replicates of the MEGA software version 11.0.10 [9] was used to construct a phylogenetic tree of selected yeast species. The identified sequences were then deposited in Genbank.

### 2.7. In Vitro Survival Test for Simulated Gastric Conditions

A total of 0.5 mL of the prepared cell suspension (10^8^ cells/mL) was transferred to a screw-cap tube containing 49.5 mL of MRSB-XOSs comprised of 0.3% (*w*/*v*) pepsin. It was then adjusted to a pH of 2.0 by the addition of 0.1 N HCl. The incubation period was carried out at 37 °C for 4 h. Samples were periodically collected for the determination of viable cells using the plate-count technique. The initial cell concentration was set to 100%.

### 2.8. In Vitro Survival Test for Simulated Intestinal Conditions

A total of 0.5 mL of the prepared cell suspension (10^8^ cells/mL) was transferred to a screw-cap tube containing 49.5 mL of MRSB-XOSs that contained 0.3% (*w*/*v*) bile salts and 0.3% (*w*/*v*) pancreatin. Incubation was carried out at 37 °C for 8 h. Samples were periodically collected for the determination of viable cells using the plate count technique. The initial cell concentration was set to 100%.

### 2.9. Cell Surface Hydrophobicity Assay

Bacterial adherence was determined by cell surface hydrophobicity [10]. Hydrophobicity is an indirect screening tool that can be used to test and select the adhesion potential of LAB into host intestinal mucosa [11]. A total of 3 mL of the bacterial suspension in PBS (A_initial_) was transferred to a glass tube (12 × 100 mm) containing 1 mL of chloroform, agitated using a vortex mixer for 2 min, and allowed to stand at 37 °C for 30 min. The optical density of the aqueous phase (A_final_) was measured at a wavelength of 600 nm. The hydrophobicity index (HPBI) was calculated using the following equation:

$$\mathrm{HPBI}\;(\%)=(1-\frac{A_{\mathrm{final}}}{A_{\mathrm{initial}}})\;\times \;100$$

### 2.10. Auto-Aggregation Assay

The auto-aggregation of probiotics is believed to be aligned with the adhesion of LAB to the intestinal epithelium [11]. A total of 3 mL of the bacterial suspension in PBS (A_initial_) was transferred to a glass tube (12 × 100 mm), vortexed for 10 s, and incubated at 37 °C for 12 h. The absorbance of the upper part of the mixture (approximately 1 mL) was measured at 600 nm (A_final_) [10]. Auto-aggregation (AA) was calculated using the following equation:

$$\mathrm{Auto-aggregation}\;(\%)=(1-\frac{A_{\mathrm{final}}}{A_{\mathrm{initial}}})\;\times \;100$$

### 2.11. Fermentation of XOSs

A total of 10% (*v*/*v*) inoculum was transferred to a screw-cap tube containing 40 mL of mMRSB-XOSs and incubated at 37 °C for 24 h. The culture was centrifuged at 17,350× *g* for 10 min at 4 °C. The clear supernatant was assigned as a crude extracellular enzyme. It was also used for the determination of the residual carbohydrate concentration, the profile of XOSs, antimicrobial activity, and short-chain fatty acids (SCFAs). Subsequently, cell pellets were washed twice with PBS solution and resuspended with the same volume of 100 mM sodium phosphate buffer at a pH of 6.5. A half-volume of the cell suspension was used as a cell-associated enzyme, while the rest was disrupted for 5 min using a sonicator. The resulting cell-free extract was designated as an intracellular enzyme.

#### 2.11.1. Investigation of Antimicrobial Activity

The antimicrobial activity of the XOSs-fermented broth obtained from the previous preparation, as described in Section 2.11, was assessed against foodborne pathogens using the agar well diffusion method [10]. The foodborne pathogens included *B*. *cereus* TISTR 747, *E*. *coli* TISTR 527, *S*. Thyphimurium TISTR 1472, and *L*. *monocytogenes* DMST 17303. 

#### 2.11.2. Determination of Xylanase and β-Xylosidase Activities

For the xylanase activity assay, 50 μL of the enzyme was reacted with 50 μL of the substrate (0.5% (*w*/*v*) xylan in 100 mM of sodium phosphate buffer, pH 6.5. The reaction was carried out at 37 °C for 10 min. Then, 100 μL of DNS solution was added to stop the reaction prior to heating at 100 °C for 10 min. After allowing the reaction to cool, 800 μL of distilled water was added to the reaction. It was then mixed, and the absorbance was measured at 540 nm. One unit (1 U) of xylanase was defined as the amount of the enzyme that released 1 μmole of reducing sugars in 1 min under assay conditions. The xylanase activity was expressed in terms of mU/mL culture.

For the β-xylosidase activity assay, 25 μL of enzyme was reacted with 225 μL of substrate (2 mM *p*-nitrophenyl xylopyranoside in sodium phosphate buffer at pH 6.5). The reaction was carried out at 37 °C for 10 min. After that, 500 μL of 2% (*w*/*v*) sodium carbonate was added to stop the reaction. Finally, the absorbance at 405 nm was measured. One unit (1 U) of β-xylosidase was defined as the amount of the enzyme that released 1 μmole of *p*-nitrophenol in 1 min under assay conditions. The β-xylosidase activity was expressed in terms of mU/mL culture.

#### 2.11.3. Thin-Layer Chromatography

TLC was performed according to a previously described method [7]. A sample of 1 μL was spotted on an aluminum silica gel plate. After applying a dryer, the plate was developed in a chamber containing *n*-butanol/ethanol/water (5:3:2 *v*/*v*/*v*). The developing procedure was conducted twice to separate XOSs and xylose better. The TLC plate was then dried and sprayed with 0.5% (*w*/*v*) thymol in a mixed solution of 5% (*v*/*v*) H_2_SO_4_ in ethanol. The spots of the carbohydrates were visualized by heating at 100 °C for 10 min.

#### 2.11.4. Determination of SCFAs

SCFAs, including acetic acid, lactic acid, propionic acid, and butyric acid, were analyzed by HPLC according to the previously described method [7].

#### 2.11.5. Antibiotic resistance

Antibiotic susceptibility was determined using Hicomb Mic test^®^ strips (HiMedia, Nashik, India) of ampicillin (Amp), vancomycin (Van), gentamicin (Gen), kanamycin (Kan), erythromycin (Ery), clindamycin (Cli), tetracycline (Tet), and chloramphenicol (Chl). Briefly, an overnight culture (approximately 10^6–^10^8^ CFU/mL) of the LAB was swabbed onto mMRSA-XOS plates. The antibiotic strip was carefully placed onto the plates. The plates were then incubated at 37 °C for 24 h. The results were expressed as susceptible, S; intermediate, I; or resistant, R, as has been established by the Clinical and Laboratory Standard Institute [12].

### 2.12. Hemolytic Activity Test

A hemolysis assay was conducted using Columbia blood agar supplemented with 5% (*v*/*v*) sheep’s blood. LAB strains were streaked on a blood agar plate and incubated at 37 °C for 48 h. Colonies that formed a clear zone and that were surrounded with a green hue were identified as β-hemolysis and α-hemolysis, respectively, while those that exhibited no clear zone were identified as γ-hemolysis.

### 2.13. Analytical Methods

All experiments were performed in duplicate. The results are presented as values of the mean ± standard deviation. Data analysis of the mean values was performed based on a full factorial complete randomized design (CRD). Briefly, data were subjected to analysis of variance (ANOVA). Multiple comparison tests were performed based on all pairwise comparisons using Tukey’s HSD test at a 95% confidence level. All analyses were carried out using the Statistix software version 8.0 (Analytical software, Tallahassee, FL, USA). A probability value of *p* < 0.05 was considered significant.

## 3. Results

### 3.1. Isolation of XOSs-Fermenting LAB

A total of 51 XOSs-fermenting LAB were recovered from 24 samples of pickled bamboo shoot products collected from the Chiang Mai, Chiang Rai, Phayao, Phrae, and Nan Provinces of Thailand. All were characterized as Gram-positive. Notably, the assumed XOSs-fermenting LAB were found in all samples, and the number of isolates varied among all samples. Nine out of 24 samples possessed more than two different colonies (Table 1). All assumed XOSs-fermenting LAB were further confirmed by their ability to ferment XOSs.

### 3.2. Selection of XOSs-Fermenting LAB

**Figure 2:**
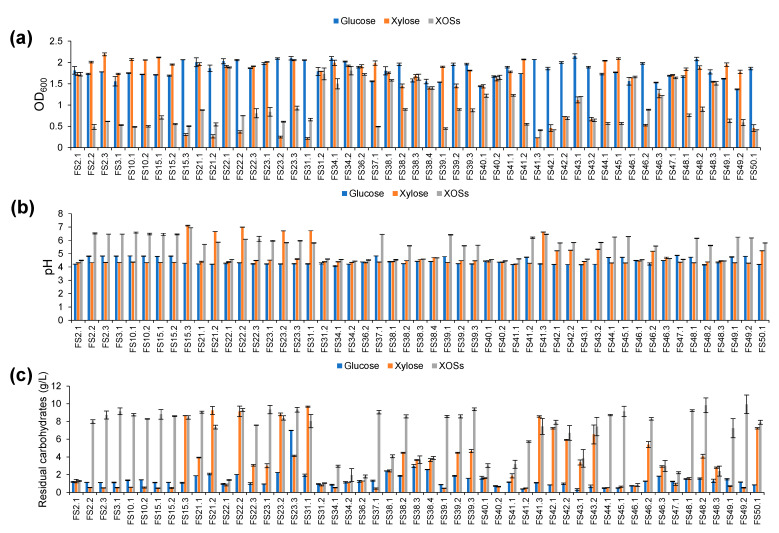
Effect of glucose, xylose, and XOSs on growth (**a**), change in pH (**b**), and residual carbohydrate (**c**) of the assumed XOSs-fermenting LAB. The reported pH is the final value.

All tested LAB were able to grow in the medium containing XOSs as the sole carbon source to different degrees of growth. Glucose, as a representative simple sugar, was used as a control, while xylose was used as the representative pentose sugar. As judged by the OD_600_ value, LAB strains that exhibited growth in XOSs with OD_600_ values greater than 1.0 and at least their growth showed equal extents with xylose and glucose, were considered as potential XOSs-fermenting LAB (Figure 2a). Among 51 strains of the assumed XOSs-fermenting LAB, 17 strains were confirmed in terms of their XOSs-fermentability. This information corresponded to a decrease in the pH value (Figure 2b) and the resulting residue carbohydrate (Figure 2c). When the carbon source was fermented by the potential XOSs-fermenting LAB, the pH value decreased from an initial value of 6.5 to a final value within a range of pH 4.0 to 4.5, while less than 4 g/L of the initial carbon source was retained.

### 3.3. Identification of XOSs-Fermenting LAB

The 16S rRNA gene sequence analysis of 17 LAB strains was performed to assess the level of diversity among potential XOSs-fermenting LAB. An online similarity analysis of all 16S rRNA gene sequences revealed that all strains were closely related to *Lv. brevis*, *P. acidilactici*, *P. pentosaceus*, *Lc. lactis*, *Lb. argentoratensis*, *Ls. fermentum*, and *Lp. plantarum*. The differentiation of *Lb. argentoratensis* and *Lp. plantarum* was performed by phylogenetic analysis according to the previously described protocol [13]. The 16S rRNA gene was deposited in GenBank under an individual accession number (Table 2).

### 3.4. Tolerance of XOSs-Fermenting LAB under Simulated GIT Conditions

Under simulated gastric conditions, all XOSs-fermenting LAB were able to survive to different degrees, with the exception of *Lc. lactis* FS38.4. After treatment for 1 h, *Lv. brevis* FS2.1, *P. acidilactici* FS38.3, *Lb. argentoratensis* FS40.1, *Ls. fermentum* FS43.1, *Lv. brevis* FS46.1, *Lp. plantarum* FS46.3, *Lv. brevis* FS47.1, and *Lp. plantarum* FS48.3 were partially inhibited, while more than 75% of the initial cell viability was retained after 2 h of incubation. Other strains were inhibited to a higher degree and retained 37% to 50% of the initial cell viability. Notably, bile salt and pancreatin had no effect on the survival of all tested XOSs-fermenting LAB (Table 3).

### 3.5. Cell Surface Hydrophobicity and Auto-Aggregation

**Figure 3:**
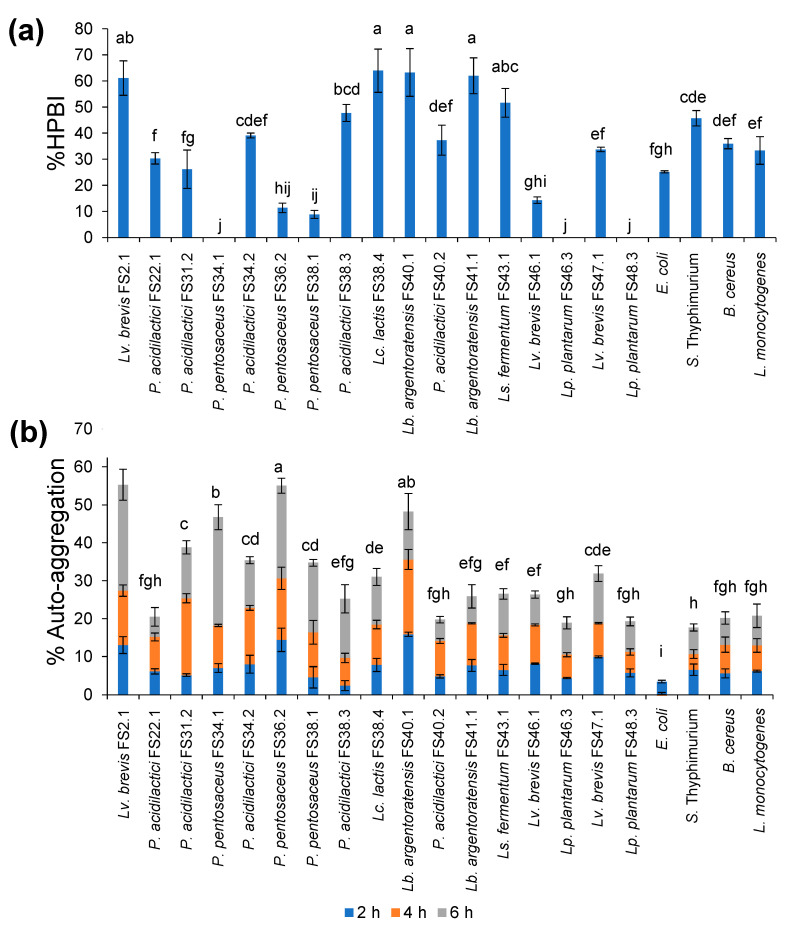
Percentages of HPBI (**a**) and AA (**b**) of the selected XOSs-fermenting LAB. Lower-case letters indicate statistical differences in values at *p* < 0.05.

Five XOS-fermenting LAB showed the highest %HPBI within a range of 50–60%. These included *Lv. brevis* FS2.1, *Lc. lactis* FS38.4, *Lb. argentoratensis* strains FS40.1 and FS41.1, and *Ls. fermentum* FS43.1. Others exhibited no significance or significantly lower %HPBI than those of the tested pathogenic bacteria (Figure 3a).

Various strains of XOSs-fermenting LAB displayed different values of %AA (Figure 3b). Eight out of seventeen strains presented significantly higher %AA than certain representative foodborne pathogens. The first four strains that demonstrated the highest %AA were *Lv. brevis* FS2.1, *P. pentosaceus* FS36.2, *Lb. argentoratensis* FS40.1, and *P. pentosaceus* FS34.1, respectively. In addition, the %AA obtained from *P. acidilactici* strains FS31.2 and FS34.2, *P. pentosaceus* FS38.1, *Lc. lactis* FS38.4, and *Lv. brevis* FS47.1 were greater than 20%. These values were higher than those of the pathogens.

### 3.6. Antimicrobial Activity

The culture supernatant obtained from XOS fermentation by all XOSs-fermenting LAB displayed antimicrobial activity against two different Gram-positive and Gram-negative pathogenic bacteria with regard to the formation of a clear zone on the agar plate. This evidence would indicate that pathogenic bacterial growth was inhibited. The wider clear zone value reflects more potential antimicrobial activity of the LAB against the tested foodborne pathogenic bacteria. The level of antimicrobial activity was indicative of both strain- and species-dependent variations (Table 4).

### 3.7. SCFAs Production

**Figure 4:**
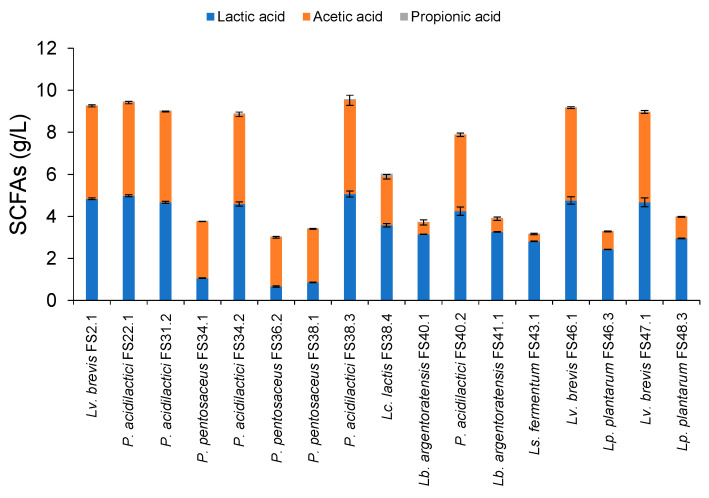
SCFAs production obtained from the selected XOSs-fermenting LAB after XOSs fermentation at 37 °C for 24 h.

SCFAs production was observed after XOSs fermentation at 37 °C for 24 h. The main SCFAs were lactic acid and acetic acid (Figure 4). Trace amounts of propionic acid were detected from all fermentation cultures. All strains of *Lv. lactis* and *P. acidilactici* produced markedly higher amounts of total SCFAs within a range of 8–9 g/L, wherein lactic acid and acetic acid were equally produced at approximately 4–4.5 g/L. The LAB that exhibited weak levels of growth on XOSs produced significantly lower levels of SCFAs than those that exhibited high amounts of growth on XOSs. This may have been associated with the degree of inefficiency in XOSs and xylose fermentation (Figure 2).

### 3.8. Antibiotic Susceptibility and Hemolytic Activity

All XOSs-fermenting LAB were susceptible to ampicillin, chloramphenicol and erythromycin, tetracycline, and clindamycin, while they were resistant to vancomycin (Table 5). Resistance against gentamycin and kanamycin was associated with species-dependent variations. Intermediately susceptible strains against gentamycin included only *P. acidilactici*, while other LAB were found to be susceptible. On one hand, all strains of *Lv. brevis* were susceptible to kanamycin. In addition, all XOSs-fermenting LAB showed no clear zone on blood agar plates, indicating γ-hemolysis.

### 3.9. Detection of Xylanolytic Enzymes

Xylanase activity was detected from the culture supernatant of all XOSs-fermenting LAB, while it was undetectable for cell suspension and cell extract. This appears to function in contrast with the β-xylosidase activity, which was found to be associated with the cells and partly found in cell extract (Table 6). Considering the residual total carbohydrate values, strains that produced both xylanase and cell-associated β-xylosidase resulted in significantly lower amounts of total carbohydrates than those that produced xylanase alone. Notably, the strain *Lb. argentoratensis* FS40.1 exhibited the highest degree of xylanase activity at 2187.2 ± 198.1 mU/mL.

**Figure 5:**
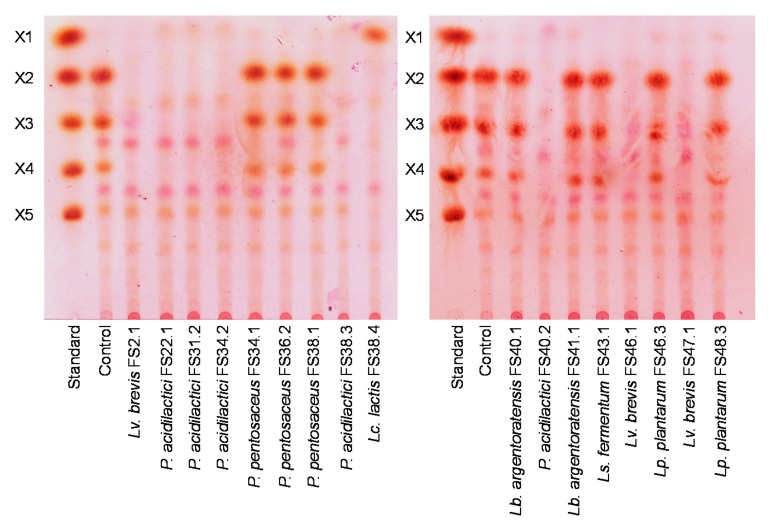
TLC chromatogram of the XOSs fermentation broth of the selected XOSs-fermenting LAB after XOSs fermentation at 37 °C for 24 h. X1 = xylose; X2 = xylobiose; X3 = xylotriose; X4 = xylotetraose; X5 = xylopentaose.

A profile of the residual XOSs is shown in Figure 5. Here, three different patterns of the retained carbohydrates were found as follows; pattern I, XOSs were clearly degraded, and xylose was effectively fermented (*Lv. brevis* strains FS2.1, FS 46.1, and FS 47.1, and *P. acidilactici* strains FS22.1, FS31.2, FS34.2, FS38.3, and FS40.2); pattern II, XOSs were clearly degraded, and residual xylose was retained (*Lc. lactis* FS38.4); pattern III, XOSs were partially degraded to shorter-chain XOSs, and trace amounts of residual xylose were retained (*P. pentosaceus* strains FS34.1, FS36.2, and FS38.1, *Lb. argentoratensis* strains FS40.1 and FS41.1, *Ls. fermentum* FS43.1, *Lp. plantarum* strains FS46.3, and FS48.3).

## 4. Discussion

Fermentation is a slow decomposition process of organic substrates that may be induced by microorganisms or enzymes in order to convert carbohydrates to alcohols or organic acids [14]. Fermented foods and beverages are associated with different traditions and cultural preferences across the various geographical areas from where they are produced, and this can result in different microbial diversities. As bamboo shoots are one of the various xylan-containing plant materials, Naw Mai Dong would be a beneficial microbial resource in the isolation of microorganisms that are associated with the degradation of xylan, as well as XOSs. The production process of Naw Mai Dong is relatively similar to that of Soidon, a non-salted acidic fermented bamboo shoot product of Manipur, India [15], and other pickled bamboo shoot products [16], but it is produced by a more simplified process than has been previously described. It is further believed that the different aspects of local wisdom that correlate with different geographical regions may be a significant point in the search for the distinct microbial diversity that is associated with each fermented product, as has been found in Phak-Gad-Dong, a pickled mustard green of northern Thailand [17]. Thus, the bacterial diversity of Naw Mai Dong may be different to that of other kinds of pickled bamboo shoots. There has been no record of the isolation of XOSs-fermenting LAB from pickled fruits and vegetables through an enrichment step. The confirmation step was established to ensure the XOSs-fermenting ability of the isolated LAB. Strains that are able to ferment XOSs, at least an equal extent with respect to the reference sugars, xylose, and glucose, could be considered XOSs-fermenting LAB. Consequently, 17 LAB strains were selected for further study. These LAB included seven species, namely *Lv. brevis*, *P. acidilactici*, *P. pentosaceus*, *Lc. lactis*, *L. argentorantensis*, *Ls. fermentum*, and *Lp. plantarum*. Two LAB species, namely *Lv. brevis* and *P. acidilactici*, are recognized as good candidates for the efficient degradation and utilization of XOSs, because their growth in glucose, xylose, and XOSs was luxuriant, while *Lc. lactis*, *Lb. argentoratensis*, *Lp. plantarum*, and *Ls. fermentum* showed moderate growth in xylose and XOSs. Remarkably, their degree of growth was weaker than that of *Lv. brevis* and *P. acidilactici*.

The selected XOSs-fermenting LAB species investigated in this study were similar to the species in the fermented bamboo shoot products of Indian origin, i.e., *Lv. brevis*, *P. pentosaceus*, and *Lp. plantarum* [15,18]. It is noteworthy to mention that *Lb. argentoratensis* is an elevated species of *Lb. plantarum* [13], yet the recovery of this species is assumed to be similar to that of *Lp. plantarum*. Although it has been well-documented that *Lc. lactis* is generally found in naturally fermented dairy products [19], it can also be found in fresh and fermented vegetables [20]. Its xylan-degrading ability has been well characterized [21]. Previously, *P. acidilactici* was isolated from various dairy products [22], malt [23], and kimchi [24]. However, the detection of this species in other ethnic pickled bamboo shoot products is still unknown, according to the results of many current studies [15,18,25]. It appears that the sampling location would limit the recovery of LAB species. Most XOSs-fermenting LAB isolated from samples collected from Chiang Mai Province were highly distributed with *Pediococcus* sp., while those isolated from samples derived from the Chiang Rai, Phayao, Phrae, and Nan Provinces were *Lactobacillus* sp.

One of the most essential characteristics of probiotic strains is their survival under GIT conditions. This attribute is required in order to confirm that the probiotic strains can pass through the GIT and reach the large intestines, where they exert a range of beneficial effects. Here, 16 strains of XOSs-fermenting LAB resisted gastric and intestinal conditions by retaining more than 40% of their initial cell viability. On the other hand, *Lc. lactis* FS38.4 lacked probiotic properties due to its inability to survive under gastric conditions. Consequently, this strain was excluded from the cell surface hydrophobicity, auto-aggregation, and antibiotic susceptibility experiments.

Cell surface hydrophobicity is defined as a nonspecific interaction in adhesion between probiotic microorganisms onto GIT epithelial cells, where they may provide prophylactic and therapeutic benefits [26]. Colonization in the epithelial cells of the large intestine and mucosal surfaces can prevent pathogenic bacteria adhesion and inflammatory reactions [27]. Auto-aggregation is defined as aggregation among LAB to form flocs and colonize the intestinal environment of the host when the cells approach harmful conditions [26]. Auto-aggregation and cell surface hydrophobicity are characteristics that provide potential advantages for microorganisms in colonizing the intestinal tract [28]. Factually, probiotics should be associated with higher values of %HPBI and %AA than pathogens. However, these attributes may or may not be correlated with one another [28]. Remarkably, *Lv. brevis* FS2.1 and *Lv. brevis* FS40.1 were the only two strains that exhibited high %auto-aggregation and %HPBI. 

The production of antimicrobial substances is an important advantage of probiotics. During growth, probiotics produce antimicrobial compounds, i.e., ethanol, fatty acids, hydrogen peroxide, and bacteriocins that can inhibit various bacterial pathogens [29]. In agreement with the findings of this study, all XOSs-fermenting LAB displayed antimicrobial activity against foodborne pathogens. In the case of *Lv. brevis* and *P. acidilactici*, it was confirmed that the inhibitory effect might have been caused by the secretion of SCFAs, namely lactic acid and acetic acid, as these species produced approximately 90% of the total SCFAs from XOSs. On the other hand, the remaining XOS-fermenting LAB species produced a low amount of total SCFAs; thus, they may have other inhibitory mechanisms i.e., bacteriocins and bacteriocin-like substances [30]. 

Antibiotics are the most effective drugs available for the treatment of bacterial infections [31]. When LAB are used as probiotics, they are known to harbor antibiotic-resistant genes that can be transferred to pathogenic bacteria and the commensal bacteria present in the gut [32], which in turn can cause significant suffering to people with pathogen infections [11]. Therefore, probiotics that are sensitive to commonly prescribed antibiotics at low concentrations have a desirable character. In addition, it is recommended that probiotics should be susceptible to at least two clinically relevant antibiotics [33]. In this study, 16 strains of XOSs-fermenting LAB were susceptible to six out of eight different tested antibiotics, and their safety was confirmed with regard to their negative hemolytic activity.

To evaluate the efficiency for XOSs fermentation, the production of a xylanolytic enzyme is of particular relevance. Efficient degradation and utilization of XOSs are affected by the degree of polymerization (DP) of XOSs while also being strain-specific. Xylanolytic enzymes associated with XOSs degradation are mostly inducible enzymes. These include β-xylosidase, α-glucuronidase, α-L-arabinosidase, and acetyl xylan esterase, as well as xylanase [34]. The XOSs used in this study were homologous XOSs with a backbone mainly composed of β-(1,4) linkages between xylose residues; thus, β-xylosidase and xylanase were the primary focus of this study. 

As far as our research is concerned, the utilization of XOSs by probiotic LAB has been extensively studied in terms of metabolic activity, enzyme activity, and transcriptomic analysis. The discovery of a coding region for xylanase and β-xylosidase was recorded in *P. acitilactici* [35], *P. pentosaceus* [36], *Lp. plantarum*, and *Lv. brevis* [34]. Previously, XOSs were proposed to be degraded by intracellular xylanase and β-xylosidase [34,37]. It has been well documented that xylanase is an endo-type enzyme that randomly catalyzes the hydrolysis of xylan to release XOSs and xylose, while β-xylosidase acts as an exo-type enzyme that catalyzes the hydrolysis of non-reducing end xylose residues from XOSs [38]. Strains that were capable of secreting xylanase resulting from random cleavages of XOSs retained shorter-chain XOSs, mainly xylobiose (X2) and xylotriose (X3). It has also been noted that all strains might be able to produce intracellular β-xylosidase, as is commonly known; however, the amount might not be enough for effective detection. On the other hand, effective levels of degradation and utilization of XOSs were found in strains that produced both xylanase and β-xylosidase. The findings of this study were slightly different from those of a number of previously discussed reports, since xylanase was detected as an extracellular enzyme, while β-xylosidase could be found in either intracellular fractions, the whole cell, or both. The profiles of XOSs obtained after XOSs fermentation support the detected xylanolytic activity. Based on these results, it has been proposed that xylanase is a key enzyme that is capable of producing shorter-chain XOSs in promoting the release of xylose with regard to the cell-associated or intracellular β-xylosidase. LAB with cell-associated β-xylosidase resulted in more considerable XOSs degradation than those that produced only intracellular enzymes. The production of extracellular xylanase is not commonly found in LAB, thus providing new insight into alternative sources of xylanase. It is important to note that not only will the ability to produce xylanolytic enzymes, but also the characteristic of xylose fermentability, effectively support proficient XOSs fermentation.

According to its overall attributes, *Lv. brevis* FS2.1 is considered the most promising strain among all XOSs-fermenting LAB. This has been determined by its resistance to GIT conditions, high values of %auto-aggregation, %cell surface hydrophobicity, antimicrobial production, and efficient degradation and utilization of XOSs. In consideration of their survival under GIT conditions and antimicrobial activity against foodborne pathogens, all 16 strains of XOSs-fermenting LAB could be used as probiotics; however, further in vivo model experiments would be necessary to confirm their health-beneficial properties.

## 5. Conclusions

This study revealed that the pickled bamboo shoot products of northern Thailand can be considered an alternative beneficial source of XOSs-fermenting LAB. Sixteen strains of the isolated LAB were confirmed to have probiotic properties according to survival under simulated GIT conditions and for their antimicrobial activity. The most promising strain was *Lv. brevis* FS2.1, as it exhibited the highest percentages of survival under GIT conditions, cell surface hydrophobicity, auto-aggregation, and efficient degradation and utilization of XOSs. Other XOSs-fermenting LAB also exhibited certain beneficial attributes, indicating their potential to be used as probiotics. It is strongly believed that the XOSs-fermenting LAB could be used for the development of synbiotic supplementation containing the approved LAB and XOSs. This would occur in conjunction with the LAB’s fermenting ability of xylose being released after the effective enzymatic degradation of XOSs was recognized as a crucial attribute in promoting the successful fermentation of xylose release after the effective enzymatic degradation of XOSs. Remarkably, xylanase was identified and classified as an extracellular enzyme, while also emerging as a potential source of XOSs-fermenting probiotic LAB. This finding may shed light on Naw Mai Dong as a promising source of xylanolytic enzyme-producing LAB. 

## Figures and Tables

**Table 1:** Sample code, sampling locations, date achieved of pickled bamboo shoot products, number of LAB isolates, and their codes.

No.	Sample Code	Subdistrict, District, Province	Location	Date Achieved	No. of Isolates	Isolate Code
1	FS2	Mae Faek Mai, San Sai, Chiang Mai	18.9950, 98.9778	8 June 2020	3	FS2.1, FS2.2, FS2.3
2	FS3	Suthep, Muang, Chiang Mai	18.7904, 98.9595	9 June 2020	1	FS3.1
3	FS10	Rim Tai, Mae Rim, Chiang Mai	18.9133, 98.9445	16 June 2020	2	FS10.1, FS10.2
4	FS15	Pa Daet, Pa Daet, Chiang Rai	19.4998, 99.9905	20 June 2020	3	FS15.1, FS15.2, FS15.3
5	FS21	Pong Tam, Chai Prakarn, Chiang Mai	19.7312, 99.1029	11 August 2020	2	FS21.1, FS21.2
6	FS22	Mae Sa, Mae Rim, Chiang Mai	18.8924, 98.6798	12 August 2020	3	FS22.1, FS22.2, FS22.3
7	FS23	Mae Sa, Mae Rim, Chiang Mai	18.8924, 98.6798	16 August 2020	3	FS23.1, FS23.2, FS23.3
8	FS31	Ki Lek, Mae Taeng, Chiang Mai	19.0781, 98.9405	16 August 2020	2	FS31.1, FS31.2
9	FS34	San Phi Suea, Mueang, Chiang Mai	18.8371, 98.9872	23 August 2020	2	FS34.1, FS34.2
10	FS36	Mae Taeng, Mae Taeng, Chiang Mai	19.0945, 98.9012	24 August 2020	1	FS36.2
11	FS37	San Phak Wan, Hang Dong, Chiang Mai	18.7227, 98.9575	25 August 2020	1	FS37.1
12	FS38	Nong Kwai, Hang Dong, Chiang Mai	18.7381, 98.9312	31 August 2020	4	FS38.1, FS38.2, FS38.3, FS38.4
13	FS39	Han Kaeo, Hang Dong, Chiang Mai	18.6535, 98.9035	31 August 2020	3	FS39.1, FS39.2, FS39.3
14	FS40	Hang Dong, Hang Dong, Chiang Mai	18.6894, 98.9036	5 September 2020	2	FS40.1, FS40.2
15	FS41	Rong Chang, Pa Daet, Chiang Rai	19.4948, 99.9414	5 September 2020	3	FS41.1, FS41.2, FS41.3
16	FS42	Pa Ngae, Pa Daet, Chiang Rai	19.5545, 99.9463	5 September 2020	2	FS42.1, FS42.2
17	FS43	Pa Daet, Pa Daet, Chiang Rai	19.5116, 99.9925	5 September 2020	2	FS43.1, FS43.2
18	FS44	Tha Wang Thong, Muang, Phayao	19.1768, 99.8932	5 September 2020	1	FS44.1
19	FS45	Wang Nuer, Wang Nuer, Lampang	19.1355, 99.6323	5 September 2020	1	FS45.1
20	FS46	Wang Nuer, Wang Nuer, Lampang	19.1355, 99.6323	5 September 2020	3	FS46.1, FS46.2, FS46.3
21	FS47	Rong Kwang, Rong Kwang, Phrae	18.3371, 100.3165	20 September 2020	1	FS47.1
22	FS48	Rong Kwang, Rong Kwang, Phrae	18.3371, 100.3165	20 September 2020	3	FS48.1, FS48.2, FS48.3
23	FS49	Klang Wiang, Wiang Sa, Nan	18.5626, 100.7466	20 September 2020	2	FS49.1, FS49.2
24	FS50	Klang Wiang, Wiang Sa, Nan	18.5626, 100.7466	20 September 2020	1	FS50.1

**Table 2:** Molecular identification of 16S rRNA gene sequences of XOSs-fermenting LAB.

Isolates	Nucleotides	% Similarity	Identification Results	Accession Number
FS2.1	1430	99.86	*Levilactobacillus brevis*	OM899733
FS22.1	1454	99.79	*Pediococcus acidilactici*	OM899734
FS31.2	1447	99.79	*Pediococcus acidilactici*	OM899735
FS34.1	1450	100.00	*Pediococcus pentosaceus*	OM899736
FS34.2	1460	99.79	*Pediococcus acidilactici*	OM899737
FS36.2	1440	100.00	*Pediococcus pentosaceus*	OM899738
FS38.1	1440	100.00	*Pediococcus pentosaceus*	OM899739
FS38.3	1440	99.79	*Pediococcus acidilactici*	OM899740
FS38.4	1420	99.93	*Lactococcus lactis*	OM899741
FS40.1	1428	99.93	*Lactobacillus argentoratensis*	OM899742
FS40.2	1440	99.79	*Pediococcus acidilactici*	OM899743
FS41.1	1440	99.86	*Lactobacillus argentoratensis*	OM899744
FS43.1	1440	99.93	*Limosilactobacillus fermentum*	OM899745
FS46.1	1430	99.79	*Levilactobacillus brevis*	OM899746
FS46.3	1424	99.93	*Lactiplantibacillus plantarum*	OM899747
FS47.1	1430	99.86	*Levilactobacillus brevis*	OM899748
FS48.3	1428	99.93	*Lactiplantibacillus plantarum*	OM899749

**Table 3:** Survival of XOSs-fermenting LAB under gastric and intestinal conditions.

Strains	Gastric Conditions			Intestinal Conditions		
	0 h	1 h	2 h	0 h	4 h	8 h
*Lv. brevis* FS2.1	100.0 ± 0.15	93.0 ± 1.70	74.5 ± 0.94	100.0 ± 0.98	103.1 ± 0.43	109.1 ± 0.38
*P. acidilactici* FS22.1	100.0 ± 0.19	85.5 ± 1.23	47.2 ± 2.16	100.0 ± 0.41	101.3 ± 0.07	104.7 ± 0.18
*P. acidilactici* FS31.2	100.0 ± 0.13	76.8 ± 0.51	46.8 ± 2.14	100.0 ± 0.37	102.5 ± 0.23	108.0 ± 0.66
*P. pentosaceus* FS34.1	100.0 ± 0.11	85.3 ± 1.78	43.0 ± 2.41	100.0 ± 0.22	101.6 ± 0.22	101.9 ± 0.23
*P. acidilactici* FS34.2	100.0 ± 0.20	84.6 ± 0.37	37.9 ± 4.12	100.0 ± 0.09	101.2 ± 0.08	106.1 ± 0.67
*P. pentosaceus* FS36.2	100.0 ± 0.30	87.3 ± 0.65	43.9 ± 2.46	100.0 ± 0.47	100.6 ± 0.19	101.4 ± 0.20
*P. pentosaceus* FS38.1	100.0 ± 0.53	82.3 ± 2.21	53.0 ± 2.21	100.0 ± 0.42	102.3 ± 0.50	103.0 ± 0.35
*P. acidilactici* FS38.3	100.0 ± 0.16	91.8 ± 0.10	78.6 ± 0.48	100.0 ± 0.29	103.0 ± 0.51	108.6 ± 0.33
*Lc. lactis* FS38.4	100.0 ± 1.34	0.0 ± 0.00	0.0 ± 0.00	100.0 ± 0.93	102.9 ± 0.11	103.7 ± 0.08
*L. argentoratensis* FS40.1	100.0 ± 1.15	77.4 ± 2.08	73.6 ± 0.27	100.0 ± 0.47	102.7 ± 0.79	108.4 ± 0.27
*P. acidilactici* FS40.2	100.0 ± 0.15	79.5 ± 1.55	40.8 ± 1.23	100.0 ± 0.48	102.7 ± 0.28	106.8 ± 0.28
*L. argentoratensis* FS41.1	100.0 ± 0.17	53.4 ± 2.36	39.0 ± 0.00	100.0 ± 0.05	104.4 ± 0.20	105.6 ± 0.38
*Ls. fermentum* FS43.1	100.0 ± 2.29	93.8 ± 0.50	91.6 ± 0.92	100.0 ± 0.95	97.9 ± 0.79	97.0 ± 1.54
*Lv. brevis* FS46.1	100.0 ± 0.48	94.2 ± 0.77	90.0 ± 0.45	100.0 ± 0.91	101.4 ± 0.37	106.3 ± 0.11
*Lp. plantarum* FS46.3	100.0 ± 0.89	95.6 ± 0.13	98.3 ± 0.90	100.0 ± 0.77	105.3 ± 0.14	105.4 ± 0.18
*Lv. brevis* FS47.1	100.0 ± 1.17	97.5 ± 0.05	84.3 ± 0.77	100.0 ± 0.08	101.0 ± 0.80	102.4 ± 0.19
*Lp. plantarum* FS48.3	100.0 ± 0.79	94.3 ± 0.16	94.0 ± 0.42	100.0 ± 0.41	108.6 ± 0.55	112.5 ± 0.68

**Table 4:** Range of clear zone (centimeter) representing antimicrobial activity against some foodborne pathogens of culture broth obtained from XOSs fermentation by XOSs-fermenting LAB.

Strains	*E. coli*	*B. cereus*	*S.* Thyphimurium	*L. monocytogenes*
*Lv. brevis* FS2.1	0.2 ± 0.0	0.4 ± 0.0	0.4 ± 0.0	1.0 ± 0.1
*P. acidilactici* FS22.1	0.7 ± 0.0	0.8 ± 0.0	0.3 ± 0.0	0.3 ± 0.0
*P. acidilactici* FS31.2	0.6 ± 0.1	0.6 ± 0.3	0.5 ± 0.1	0.5 ± 0.1
*P. pentosaceus* FS34.1	0.8 ± 0.1	1.0 ± 0.1	1.0 ± 0.3	1.0 ± 0.1
*P. acidilactici* FS34.2	0.6 ± 0.0	0.7 ± 0.0	0.4 ± 0.1	0.4 ± 0.0
*P. pentosaceus* FS36.2	0.4 ± 0.1	1.1 ± 0.0	0.7 ± 0.4	0.7 ± 0.1
*P. pentosaceus* FS38.1	0.4 ± 0.1	0.9 ± 0.0	1.5 ± 0.2	0.5 ± 0.1
*P. acidilactici* FS38.3	0.2 ± 0.1	1.0 ± 0.1	1.3 ± 0.4	0.3 ± 0.0
*Lc. lactis* FS38.4	0.5 ± 0.1	1.0 ± 0.1	0.8 ± 0.1	0.4 ± 0.1
*L. argentorantensis* FS40.1	0.6 ± 0.1	1.0 ± 0.1	0.6 ± 0.1	0.5 ± 0.1
*P. acidilactici* FS40.2	0.6 ± 0.1	0.7 ± 0.1	0.9 ± 0.1	0.8 ± 0.1
*L. argentorantensis* FS41.1	0.8 ± 0.1	0.3 ± 0.1	0.5 ± 0.1	0.8 ± 0.1
*Ls. fermentum* FS43.1	0.7 ± 0.0	0.5 ± 0.1	0.6 ± 0.1	0.7 ± 0.1
*Lv. brevis* FS46.1	0.9 ± 0.0	0.7 ± 0.1	0.9 ± 0.0	0.9 ± 0.0
*Lp. plantarum* FS46.3	0.6 ± 0.1	0.6 ± 0.1	0.9 ± 0.0	0.8 ± 0.1
*Lv. brevis* FS47.1	0.6 ± 0.0	0.7 ± 0.1	0.9 ± 0.0	0.9 ± 0.0
*Lp. plantarum* FS48.3	1.0 ± 0.1	1.0 ± 0.2	1.0 ± 0.1	1.1 ± 0.1

**Table 5:** Antibiotic susceptibility test of selected XOSs-fermenting LAB.

Strains	Amp	Chl	Ery	Gen	Van	Kan	Tet	Cli
*Lv. brevis* FS2.1	S	S	S	S	R	S	S	S
*P. acidilactici* FS22.1	S	S	S	I	R	R	S	S
*P. acidilactici* FS31.2	S	S	S	I	R	R	S	S
*P. pentosaceus* FS34.1	S	S	S	I	R	R	S	S
*P. acidilactici* FS34.2	S	S	S	S	R	R	S	S
*P. pentosaceus* FS36.2	S	S	S	S	R	R	S	S
*P. pentosaceus* FS38.1	S	S	S	S	R	R	S	S
*P. acidilactici* FS38.3	S	S	S	I	R	R	S	S
*L. argentoratensis* FS40.1	S	S	S	S	R	R	S	S
*P. acidilactici* FS40.2	S	S	S	I	R	R	S	S
*L. argentoratensis* FS41.1	S	S	S	S	R	R	S	S
*Ls. fermentum* FS43.1	S	S	S	S	R	R	S	S
*Lc. brevis* FS46.1	S	S	S	S	R	S	S	S
*Lp. plantarum* FS46.3	S	S	S	S	R	R	S	S
*Lv. brevis* FS47.1	S	S	S	S	R	S	S	I
*Lp. plantarum* FS48.3	S	S	S	S	R	R	S	S

S = susceptible; I = intermediate; R = Resistant.

**Table 6:** β-xylosidase and xylanase activities of XOSs-fermenting LAB after being grown in MRSB-XOSs at 37 °C for 24 h.

Strains	β-Xylosidase (mU/mL)			Xylanase (mU/mL)		
	Extracellular	Cell-Associated	Intracellular	Extracellular	Cell-Associated	Intracellular
*Lv. brevis* FS2.1	ND	29.4 ± 3.1	ND	320.8 ± 17.3	ND	ND
*P. acidilactici* FS22.1	ND	79.2 ± 1.8	9.3 ± 0.1	280.9 ± 76.6	ND	ND
*P. acidilactici* FS31.2	ND	110.2 ± 0.5	12.5 ± 0.7	450.5 ± 7.2	ND	ND
*P. pentosaceus* FS34.1	ND	ND	ND	871.4 ± 10.1	ND	ND
*P. acidilactici* FS34.2	ND	122 ± 7.2	9.8 ± 0.4	278.9 ± 67.9	ND	ND
*P. pentosaceus* FS36.2	ND	ND	ND	545.5 ± 52.0	ND	ND
*P. pentosaceus* FS38.1	ND	ND	ND	476.1± 89.6	ND	ND
*P. acidilactici* FS38.3	ND	106.5 ± 3.5	5.8 ± 0.3	256.4 ± 7.2	ND	ND
*Lc. lactis* FS38.4	ND	103.6 ± 3.8	31.2 ± 0.1	524.1 ± 70.8	ND	ND
*Lb. argentoratensis* FS40.1	ND	ND	ND	2187.2 ± 198.1	ND	ND
*P. acidilactici* FS40.2	ND	151.5 ± 28.4	6.6 ± 0.6	422.9 ± 23.1	ND	ND
*Lb. argentoratensis* FS41.1	ND	ND	ND	673.2 ± 79.5	ND	ND
*Ls. fermentum* FS43.1	ND	ND	5.1 ± 0.1	602.7 ± 23.1	ND	ND
*Lv. brevis* FS46.1	ND	54.0 ± 3.0	ND	279.9 ± 28.9	ND	ND
*Lp. plantarum* FS46.3	ND	ND	20.3 ± 0.9	561.9 ± 46.2	ND	ND
*Lv. brevis* FS47.1	ND	67.9 ± 7.4	24.5 ± 1.1	446.4 ± 85.2	ND	ND
*Lp. plantarum* FS48.3	ND	ND	ND	578.2 ± 60.7	ND	ND

Note: The term “extracellular” refers to extracellular enzymes that were obtained from the cell-free supernatant of the XOSs fermentation culture; “cell-associated” refers to cell-associated enzymes that were obtained from cell pellets after collecting cell-free supernatants; “intracellular” refers to intracellular enzymes that were obtained from the cell extracts of the cell pellet. ND refers to not detected.

## Data Availability

Not applicable.
